# Semantic Segmentation of Intralobular and Extralobular Tissue from Liver Scaffold H&E Images

**DOI:** 10.3390/s20247063

**Published:** 2020-12-10

**Authors:** Miroslav Jirik, Ivan Gruber, Vladimira Moulisova, Claudia Schindler, Lenka Cervenkova, Richard Palek, Jachym Rosendorf, Janine Arlt, Lukas Bolek, Jiri Dejmek, Uta Dahmen, Milos Zelezny, Vaclav Liska

**Affiliations:** 1 NTIS—New Technologies for the Information Society, Faculty of Applied Sciences, University of West Bohemia, 301 00 Pilsen, Czech Republic; grubiv@ntis.zcu.cz (I.G.); zelezny@kky.zcu.cz (M.Z.); 2 Biomedical Center, Faculty of Medicine in Pilsen, Charles University, 323 00 Pilsen, Czech Republic; Vladimira.Moulisova@lfp.cuni.cz (V.M.); Lenka.Cervenkova@lfp.cuni.cz (L.C.); palekr@fnplzen.cz (R.P.); rosendorfj@fnplzen.cz (J.R.); Lukas.Bolek@lfp.cuni.cz (L.B.); Jiri.Dejmek@lfp.cuni.cz (J.D.); liskav@fnplzen.cz (V.L.); 3 Experimental Transplantation Surgery Department, Universitätsklinikum Jena, 07743 Jena, Germany; Claudia.Schindler@med.uni-jena.de (C.S.); janine.arlt@med.uni-jena.de (J.A.); Uta.Dahmen@med.uni-jena.de (U.D.); 4 Department of Surgery, University Hospital and Faculty of Medicine in Pilsen, Charles University, 323 00 Pilsen, Czech Republic

**Keywords:** H&E, decellularization, liver, tissue engineering, semantic segmentation, convolutional neural networks

## Abstract

Decellularized tissue is an important source for biological tissue engineering. Evaluation of the quality of decellularized tissue is performed using scanned images of hematoxylin-eosin stained (H&E) tissue sections and is usually dependent on the observer. The first step in creating a tool for the assessment of the quality of the liver scaffold without observer bias is the automatic segmentation of the whole slide image into three classes: the background, intralobular area, and extralobular area. Such segmentation enables to perform the texture analysis in the intralobular area of the liver scaffold, which is crucial part in the recellularization procedure. Existing semi-automatic methods for general segmentation (i.e., thresholding, watershed, etc.) do not meet the quality requirements. Moreover, there are no methods available to solve this task automatically. Given the low amount of training data, we proposed a two-stage method. The first stage is based on classification of simple hand-crafted descriptors of the pixels and their neighborhoods. This method is trained on partially annotated data. Its outputs are used for training of the second-stage approach, which is based on a convolutional neural network (CNN). Our architecture inspired by U-Net reaches very promising results, despite a very low amount of the training data. We provide qualitative and quantitative data for both stages. With the best training setup, we reach 90.70% recognition accuracy.

## 1. Introduction

Decellularized tissue scaffolds consisting of extracellular matrix proteins after complete cell removal represent natural three-dimensional matrices with great potential in tissue engineering [1,2]. Recellularization of the decellularized scaffold can be used for in vitro engineering of artificial organs [3,4], providing an alternative strategy to other methods such as cell repopulation of synthetic matrices [5] or growing chimeric organs in genetically altered animals [6].

Nevertheless, despite research efforts, the construction of liver tissue in vitro remains very challenging. The quality of decellularized scaffold is crucial for the initial cell-scaffold interaction [7,8], and thus determines the success of the cell repopulation process. However, the assessment of the scaffold quality prior to recellularization represents one of the remaining problems to be solved. The assessment criteria available are very fragmented and concentrate mainly on bulk properties. Morphological evaluation is mostly qualitative and rather superficial [9,10].

The Whole Slide Scan microscopy (WSS) has been widely used in last years. It allows to study and archive detail images of whole samples. The image processing techniques allow to design efficient semiautomatic and automatic procedures for quantitative analysis. The general algorithms available in free software can be often successfully used to solve simple tasks. In the paper [11], the authors used ImageJ application based on the Gray Level Co-occurence Matrix and Run-Length Matrix [12] to analyze liver fibrosis in H&E images. In more complex tasks, the use of an image processing tool and using a scripting language might be necessary. In [13], the Matlab software with its script language was used for quantitative analysis of cells and tissues. The most challenging tasks require the most advanced algorithms. The convolutional neural networks introduced by LeCun in [14,15] have promising results also in WSS microscopy. The most common tasks are image classification and image segmentation. The convolutional neural network-based approach to solve this problem for nuclei and cells can be found in [16].

The first method for the quantitative evaluation of the structure quality with respect to particular liver scaffold features such as intralobular sinusoidal vessel structures was introduced in [8]. However, this method requires an initial user input thus it is observer dependent. The first step in creating an observer independent and reproducible evaluation method of the scaffold structure quality is the semantic segmentation into three classes: background, intralobular area, and extralobular area.

Due to the neural networks improvements in recent years, most hand-crafted feature descriptors for semantic segmentation, if enough data are available, become obsolete. However, a suitable dataset with liver tissues does not exist and the creation of a new one includes per-pixel labels of high-resolution data which is very time demanding and costly.

Therefore, in this paper, we propose a two-stage method. In the first stage we utilize Naive Bayes classifier [17] trained on a simple texture descriptor. The outputs of this classifier we utilize as training data for the convolutional neural network.

The main contributions of this paper are the following:

1 We introduce a two-stage method for semantic segmentation of liver scaffold hematoxylin-eosin (H&E) stained section images. In the first stage, we train the Naive Bayes classifier on simple texture descriptors. In the second stage, we utilize the classifier’s outputs as training data for U-Net-based convolutional neural network.
2 We compare the single-stage approach with the two-stage method on a small subset of manually annotated data with the two-stage method reaching superior results.

## 2. Materials and Methods

### 2.1. Scaffold Sample Preparation

After the explantation from domestic pigs (Sus scrofa), the liver was decellularized by perfusion with detergent solutions (1% Triton X-100, 1% SDS) via the portal vein and hepatic artery, and finally washed with saline using a system of peristaltic pumps (Masterflex L/S, Cole-Palmer, Vernon Hills, IL, USA). Scaffold samples were fixed in 10% buffered formalin, embedded in paraffin, and eventually cut on a microtome in 4 µm thick sections. The tissues were taken with ethical approval from the Ministry of Education of the Czech Republic (no. MSMT-4428/2018-2).

### 2.2. Histological Staining and Imaging

Histological sections were mounted on glass slides, deparaffinized, and subjected to hematoxylin-eosin staining resulting in blue stained nuclei and pink stained cytoplasm. Whole slide scans were produced using Nanozoomer 2.0HT Digital Slide Scanner (Hamamatsu, Hamamatsu City, Japan). The source lens used for data acquisition was 40×. The typical size of the source area was about 15×10 mm. The resolution of the images is 227 nm per pixel. The size of an uncompressed image data was 7 to 19 GB.

### 2.3. Image Processing

The input image of H&E stained scaffolds is described by selected texture features. As a result of the small amount of training data and the lack of full image annotation we used a two-stage method. In the first stage, the training set of partially annotated images was used. This classifier is then used per-pixel for the WSS segmentation. To increase accuracy, the classifier is trained based on a simple annotation for a particular image. Thus, the obtained segmentations are used in the second stage to train a convolutional neural network that does not require further adjustment.

### 2.4. Preprocessing and Data Annotation

**Figure 1:**
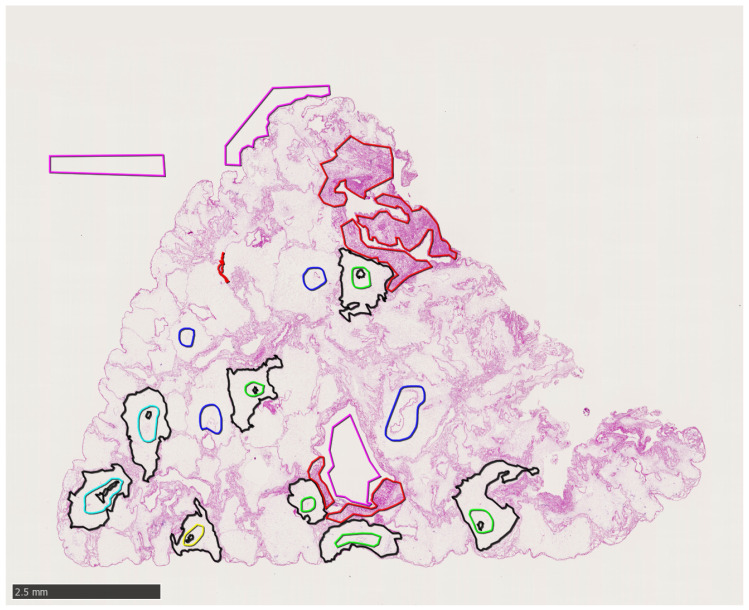
Example of partially annotated H&E Whole Slide Scan (WSS). The manually selected extralobular area is labeled in red. The magenta delineation shows the scan background and the intralobular area is annotated in black. The green, cyan, blue, and yellow annotation represents the rough delineation of the central vein.

WSS data are stored in NDPI file format and partial annotations are stored in NDPA format. Background, intralobular, and extralobular areas are annotated by with magenta, black, and red color, respectively (see Figure 1). The area with the particular type of tissue is selected by drawing a polygon. With this procedure few representative parts of the image were picked. The full annotation of the whole slide image was not generated due to large time demands. Annotations were produced by an operator supervised by a tissue engineering expert.

Based on metadata, the pixel size for each layer from the pyramid representation of the NDPI file format was extracted. The vertices of the annotation polygons were recalculated to the proper resolution. A 10 µm pixel bitmap is created from the pyramid representation of NDPI files. The image was then divided into tiles of 255×255 px for easier processing.

### 2.5. Handcrafted Texture Feature Segmentation (HCTFS)

**Figure 2:**
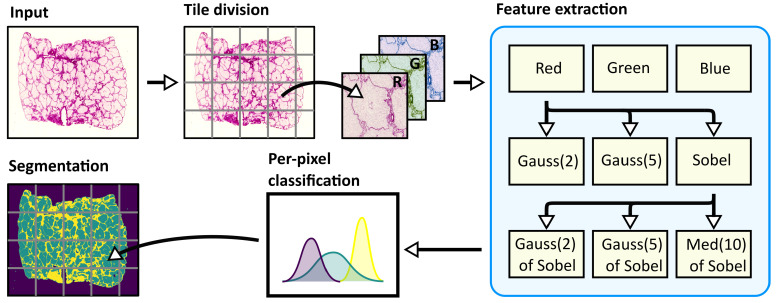
Handcrafted Texture Feature Segmentation algorithm. Input H&E stained image is divided into tiles. Each tile is processed separately. Red (R), Green (G), and Blue (B) image channels are used as first features. The Sobel filter and the Gaussian smoothing with the standard deviation of 2 pixels and 5 pixels (Gauss(2)) and Gauss(5)) are applied to the Red channel. The output of the Sobel filter is used to calculate two features based on the Gaussian of the Sobel filter with a standard deviation of 2 pixels and 5 pixels (Gauss(2) of Sobel) and the median of Sobel with neighborhood with size 10(Med(10) of Sobel). These features are used for image segmentation based on per-pixel classification.

**Figure 3:**
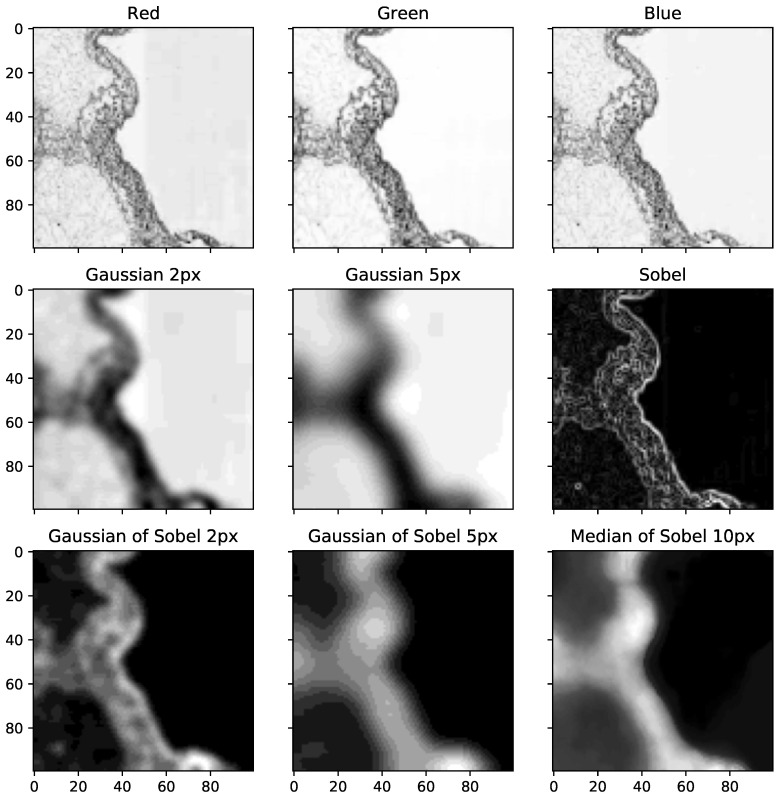
Features used for per-pixel Handcrafted Texture Feature Segmentation (HCTFS). In each subfigure, the intralobular, extralobular, and empty areas are on the left, middle (the vertical structure), and right, respectively. Red, Green, and Blue image channels are in the first row. The Gaussian smoothings with the standard deviation of 2 pixels and 5 pixels together with the Sobel filter are in the second row. The Gaussian of the Sobel filter with a standard deviation of 2 pixels and 5 pixels are in the third row. The last feature in the figure is the median of the Sobel filter with a neighborhood of size 10.

The texture features were designed to describe the pixel intensity and the neighborhood texture. We started from our formerly designed method for scaffold texture segmentation [8] and extended the feature vector to better distinguish the differences between the “background” and the “intralobular” area. The flow-chart of the algorithm can be seen in Figure 2. To keep the computation demands low, the texture features are as simple as possible. The first three features originate from RGB intensity. This takes into account the color information in the H&E stained scaffold images. Only the red channel, which is strongly correlated with other color channels, is used in the calculation of other features. The next two features are obtained by a Gaussian filter [18] with a Standard Deviation for the Gaussian kernel of 2 and 5 pixels. The Sobel filter [19] is used to describe the local discontinuity in the image. The Sobel filter response at the pixel location is used as one feature. The information from the neighborhood discontinuity is generated by the Gaussian Response Filter of the Sobel filter with a standard deviation of 2 and 5 pixels. The last feature is a median of the neighborhood of 10 pixels in diameter. The responses of each feature extractor can be found in Figure 3.

The features obtained from partially annotated areas of the image are then used to train the Gaussian Naive Bayes Classifier. The studies of the classifier can be found in the paper [20,21]. The *scikit-learn* implementation was used [22] for our experiments. The annotations were performed to distinguish the three following classes: background, intralobular areas, and extralobular areas. The classifier was pre-trained on a general dataset and then used for per-pixel segmentation. Before each use, it is additionally trained using target image data and available partial annotations for that image.

### 2.6. Fully-Convolutional Neural Network

The second tested method inspired by [23,24,25] is built upon a feed-forward fully-convolutional neural network (CNN), with an encoder–decoder structure. Based on our previous research [26], we believe that such a structure is perfectly suitable for semantic segmentation tasks. Firstly, the encoder compresses the data from raw image pixels on the input into a feature vector representation. Secondly, based on the feature vector, the decoder produces output maps with the same size as the input. One map is produced for each class, i.e., our network produces three maps in total.

Our architecture is based on U-Net [24], however, we have made a few minor changes. Firstly, our architecture also utilizes skip connections between corresponding layers of encoder and decoder, however, unlike skip connections in the original implementation of U-Net, our skip connections are implemented as element-wise additional. Secondly, due to the relatively small amount of training data, we employ a much smaller architecture to prevent overfitting. To be more specific, our architecture called UNet-Mini uses only 128k parameters, whereas the original implementation of U-Net uses over 17M parameters. Our encoder, and decoder are composed of only four (de)convolutional layers with 16, 32, 64, and 64 number of kernels, respectively, kernel size ks=3×3 and stride s=1.

**Figure 4:**
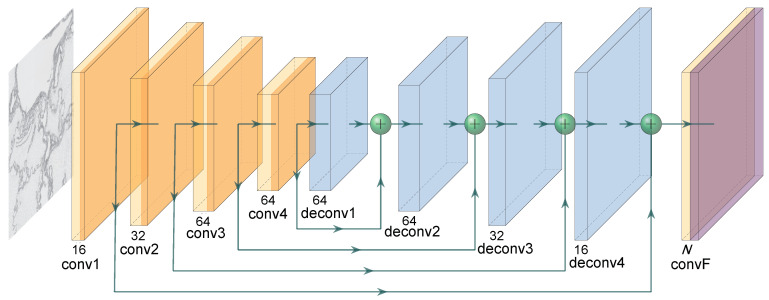
Structure of UNet-Mini architecture. The encoder is composed of four convolutional layers, each followed by batch normalization (BN) and the ReLU activation function. The decoder mirrors this structure. *N* in the last convolutional layer convF of the decoder represents the number of classes (i.e., 3). conF is followed by the Softmax activation.

Apart from these differences, our architecture follows a standard setup of (de)convolution followed by batch normalization and the ReLU activation. Four deconvolutions in decoder are followed by the convolution with kernel size ks=1×1 and stride s=1. This layer performs a classification task, therefore, it utilizes the classical Softmax activation function. The detailed description of the architecture can be found in Figure 4.

The neural network is implemented and trained in Python using Chainer deep learning framework [27,28]. Experimental settings and results can be found in Section 3.2.

## 3. Experiments and Results

### 3.1. Handcrafted Texture Feature Segmentation

**Figure 5:**
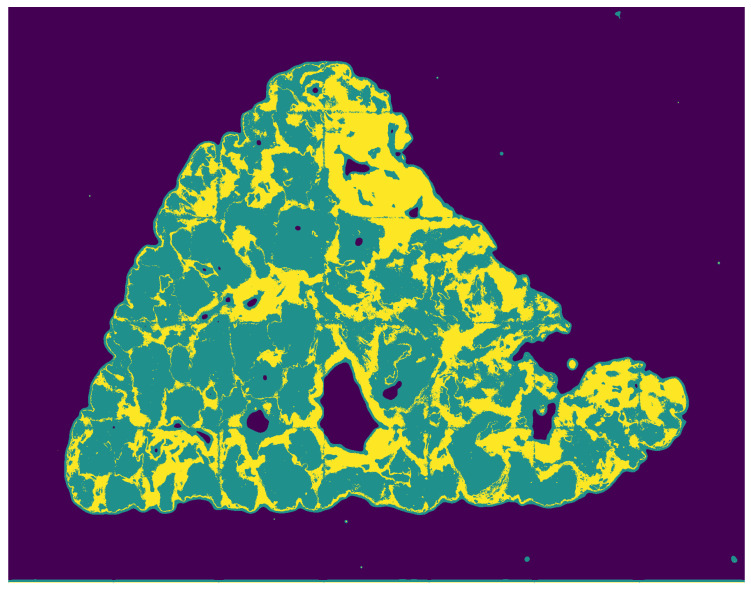
Output of the handcrafted texture feature-based segmentation. The background class is in dark purple, the intralobular area is represented by teal color, and the extralobular area is in yellow.

To train the first stage classifier in Handcrafted Texture Feature Segmentation, we used a dataset that contained 60 different areas of 8 WSS. This pre-trained classifier with small additional annotation for every image was then used to produce 33 WSS segmentations for the second stage based on CNN. The first stage segmentation output can be seen in Figure 5.

### 3.2. Semantic Segmentation via CNN

Generally, a huge amount of data is necessary for network training. For this initial experiment, we used only 33 WSS (with average resolution approximately 3000 × 2000 pixels) without any original labels. The annotations resulting from the HCTFS of the individual scans were then utilized as the labels. We believe our network should handle occasional mislabels of the HCTFS, learn the correct structure for each class, and outperform the first method.

The data were converted to gray-scale and split into three subsets—training (25 scans), development (4 scans), and testing (4 scans) set. Considering the size of scans, we decided to cut each of them into the crops of the size of 224 × 224 pixels with 100 pixels overlay. Furthermore, to produce more training data, we resized each scan to half of the original resolution and repeated the whole cutting process. This process was repeated two times in total. In the last step, we resized the original scan to the size of 224 × 224 pixels. Thanks to this process, we got 11,384 training images, 2739 development images, and 2425 testing images. Such amount of data represents still quite a small data set for the training of the neural network. To overcome this problem and improve the network’s robustness, we also used data augmentations. To be more specific, each image crop was modified with a random number of augmentations. The possible augmentations were the following: horizontal flip, vertical flip, white noise, and Gaussian blur. This process was repeated three times for each image crop. This leads to 45,536 training images in total. All the pixel values were normalized from 0 to 1.

UNet-Mini is trained for the semantic segmentation of an input image into one of the three following classes: intralobular, extralobular, and background. The Adam optimization method [29] with standard parameters setup and also standard SGD optimizer with a starting learning rate l=0.01 and step decay d=0.1 every 10 epochs were the hyperparameters we used for updating UNet-Mini’s parameters. In both cases, we use the cross-entropy loss for the network training and mini-batch size 32. The training is stopped after 35 epochs. We used 1 GPU NVidia 1080Ti for training.

Both optimizers reach comparable results, with the best recognition accuracy of 92.35% on the development set. It is necessary to note that the reached accuracy is calculated by comparing the network results with the results from HCTFS. As it was already mentioned, the HCTFS’s results contain some mislabels, therefore, our goal was not to completely replicate the original results, but to filter out these mistakes and to learn to segment the scans more precisely.

To objectively compare both methods, we manually label additional ground-truth data patch on the original images. The resulting images can be found in Table 1. UNet-Mini overcomes the HCTFS method by more than 4% on both sets. This means that UNet-Mini learns to generalize better than the original method despite incorrect data in the training set. Plus, UNet-Mini does not need any additional image specific labels.

**Figure 6:**
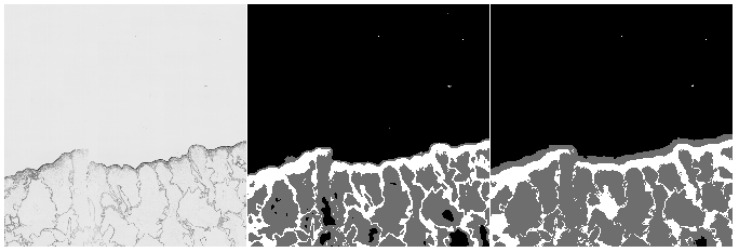
Example of semantic segmentation, where the neural network reached better results. The original image (on the **left**), the result from the HCTFS (in the **middle**), and the results from the neural network (on the **right**).

**Figure 7:**
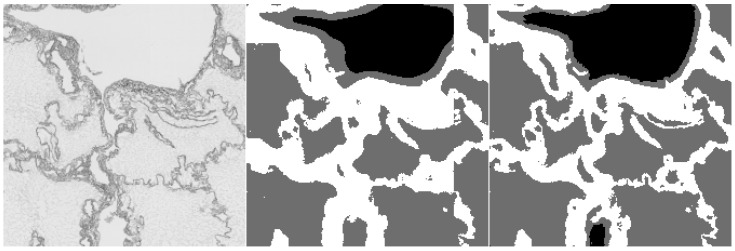
Example of semantic segmentation, where the neural network reached better results. The original image (on the **left**), the result from the HCTFS (in the **middle**), and the results from the neural network (on the **right**).

**Figure 8:**
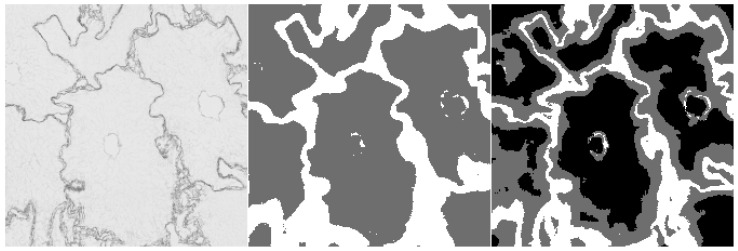
Example of semantic segmentation, where the neural network reached worse results. The original image (on the **left**), the result from the HCTFS (in the **middle**), and the results from the neural network (on the **right**).

**Figure 9:**
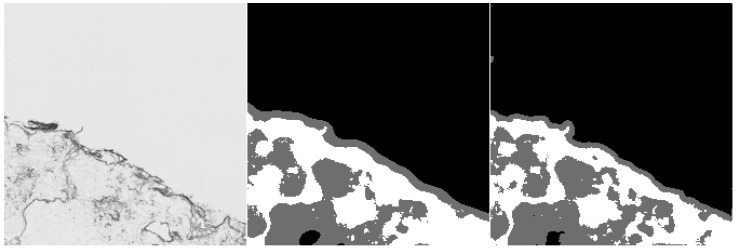
Example of semantic segmentation, where the neural network reached comparable results. The original image (on the **left**), the result from the HCTFS (in the **middle**), and the results from the neural network (on the **right**).

Furthermore, we provide examples of qualitative results comparing both methods. Figure 6 and Figure 7 show the results, where the UNet-Mini corrected or partially corrected the original mistakes in labels. On the other hand, an example of obvious mislabels made by the network can be found in Figure 8. Finally, Figure 9 provides an example of equally good results from both tested methods.

## 4. Discussion

The scaffold function is directly linked to its structure [30]. Current approaches to analyze scaffold quality include the qualification of the residual DNA content, the amount (or ratio) of structural proteins such as collagen I, collagen IV, laminin, fibronectin, or elastin, and presence of glycosaminoglycans [31,32].

The morphological assessment consists of subjective evaluation of scaffold structure preservation which is supposed to be as close to the native liver structure as possible. H&E staining represents a fast and simple histological method to visualize the scaffold structure as well as cell removal from samples. The typical structural unit of the liver is a lobule, ideally a hexagonally shaped structure with intralobular space occupied by sinusoidal vessels surrounded by hepatocytes. The scaffold consists of the extracellular matrix of the vessel walls forming conduits, empty inter-sinusoidal space after the removal of hepatocytes, and interlobular septa formed by thick protein fibers.

The presence and distribution of individual structural proteins is usually confirmed by immunohistochemistry representing more time and cost consuming method. The ultrastructure can be visualized by scanning electron microscopy; however, the cost and extended time spent during sample processing makes this powerfull technique not always available. Scaffold images obtained by any of these methods have a potential to be quantitatively analyzed. However, for the development of a new quantitative method, we selected H&E stained images. They can be produced in a fast and easy way while still carrying the information necessary to evaluate structural integrity of the scaffold.

The segmentation of liver scaffold from H&E stained image based on handcrafted texture features works well in the interactive mode where additional partial segmentation of a particular image is given. Without additional per image classifier training, the segmentation algorithm provides unstable results. This makes it dependent on the manual annotation of each examined image. Considering the very promising results that we have reached in our initial experiments, we would like to further investigate possible usage of semantic segmentation via neural networks. In our future research, we would like to extend our training set with additional slides. Moreover, we would like to perform extensive testing of other neural network architectures.

## 5. Conclusions

The first step in the decellularized liver analysis can be successfully represented by the whole slide segmentation. Due to the lack of completely annotated WSS, we designed a two-state solution. The first stage is segmentation based on hand-crafted features that are trained using partially annotated WSS. The second stage uses CNN with a U-Net scheme. The two-stage approach has proved to be useful to compensate the lack of training data, and reaches semantic segmentation accuracy over 90% and overcomes the handcrafted features by more than 4%. In our future work, firstly, we would like to enrich our dataset. Especially images obtained using different scanners are very desirable because such data can provide a classifier bigger robustness and better generalization capacity. Secondly, with more data, we believe, utilizing more complex neural network architecture would be possible. We also plan to use the suggested algorithm in the open-source application for the scaffold tissue evaluation.

## Figures and Tables

**Table 1:** Comparison of classification recognition rates. Bold font indicates best results.

Method	Dev Set	Test Set
HCTFS	86.47%	86.51%
UNet-Mini	**90.87**%	**90.67**%

## Abbreviations

The following abbreviations are used in this manuscript:

CNN	Convolutional Neural Network
H&E	Hematoxylin-eosin-staining
WSS	Whole Slide Scan
HCTFS	Handcrafted Texture Feature Segmentation
